# Schwannoma with an uncommon anal location

**DOI:** 10.3892/ol.2014.2459

**Published:** 2014-08-19

**Authors:** JIYONG PAN, HUIRONG JING, XIAOFENG TIAN, ZHE FAN

**Affiliations:** 1 Department of General Surgery, The Third People’s Hospital of Dalian, Dalian, Liaoning 116000, P.R. China; 2 Department of General Surgery, The Second Hospital of Dalian Medical University, Dalian, Liaoning 116000, P.R. China

**Keywords:** schwannoma, anal, benign, case study

## Abstract

Schwannomas are slow-growing mesenchymal neoplasms that arise from Schwann cells with low malignant potential. These uncommon neoplasms are nerve sheath tumors that arise at almost any anatomical site. The majority of schwannomas are benign, and few are malignant. The current study presents the rare case of an anal schwannoma that was successfully treated by surgery; there are few such cases previously reported in the literature. The patient was admitted to hospital following the identification of a mass incidentally. The tumor was so large that it compressed the tissue around it, although no symptoms were caused. The pre-operative clinical diagnosis was inconclusive in this case, and a final diagnosis was established based on radiographic and histopathological examination. The current study aimed to provide a possible differential diagnosis for such anally-located masses.

## Introduction

Schwannomas are derived from Schwann cells (1). More than 90% of schwannomas are benign. They often have a single place of origin and 10% have multiple locations of origin. Schwannomas often arise in the head and neck (~25–40%), but are rarely located in the retroperitoneum (6% of primary retroperitoneal tumors) (1,2). These tumors, although rare, should be considered in the differential diagnosis of slow-growing masses of an anal location, such as lipomyomas. The growth of these masses occasionally causes displacement and compression of the nerve of origin, resulting in clinical signs and symptoms (3). The tumors can develop in either of the two genders and there is no age predilection (4). Surgical excision is the treatment of choice for schwannomas (3). In the present study, the case of a patient with an anal schwannoma is reported, where the pre-operative clinical diagnosis was inconclusive and the final diagnosis was established based on radiographic and histopathological examination. Written informed consent was obtained from the patient.

## Case report

**Figure 1:**
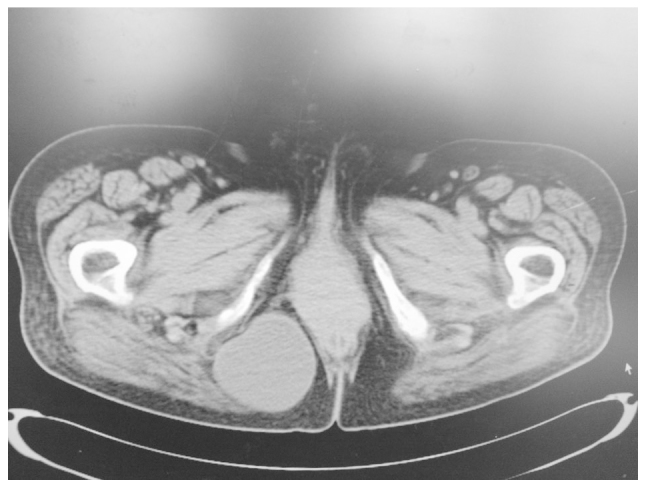
Computed tomography scan showing the location of the tumor beside the anus, and the appreciable affect on its normal structure.

**Figure 2:**
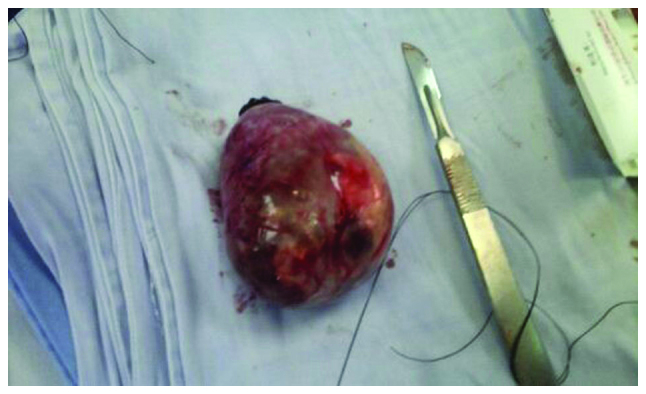
Image of the 6×8-cm resected cystic tumor.

A 66-year-old female was admitted to the Third People’s Hospital of Dalian (Dalian, China) due to a right anal mass that had been present for several weeks. The patient experienced no pain, difficulty in defecation or other symptoms. A physical examination revealed no unusual findings, with the exception of 10×7-cm, tenacious, mobile and smooth mass that could be palpated when in the knee-chest decubitus position. Computed tomography (CT) scans showed that the tumor, which had uniform density and a clear tunica, was located under a fat layer and inside of the gluteus maximus (Fig. 1). The results of blood and kidney function tests were normal and the patient underwent surgery to completely excise the tumor, with no postoperative complications. The pathological analysis reported a 6×8-cm cystic tumor, which was full of a gelatinous material (Fig. 2). Microscopically, the tumor was diagnosed as a schwannoma with cystic change. Immunohistochemistry revealed that the tumor was S-100-positive. The patient was followed-up for one year and no disease recurrence was identified.

## Discussion

Schwannomas are nerve sheath tumors that arise from almost any anatomical site, but particularly in the peripheral, cranial or visceral nerves (1). Schwannomas can be found not only in children, but in adults also.

The majority of schwannomas are benign, and few are malignant. Malignant schwannomas are usually large, infiltrating and characterized histologically by perineural and intraneural spread; in the present study, mainly benign schwannomas are discussed. Schwannomas have the characteristics of a slow growth rate, no invasion and a low rate of transformation or recurrence following resection (1).

The site of the schwannoma may vary, however, the tumors mostly occur in the neck, head and tendon sheaths of the limbs. There has also been a study reporting schwannoma of the abdomen (3).

Unusual locations, such as the anus, are rarely reported (6). In the present study, a rare mass is reported, which was believed to originate from the cranial nerves due to its location under the deep skin layer.

CT and magnetic resonance imaging (MRI) were more beneficial for the identification of characteristics of the mass, although obviously distinct in clinical features and histological appearance; the pre-operative diagnosis of a schwannoma is not easy owing to a lack of distinguishing features. It is possible to distinguish between benign and malignant schwannomas by imaging techniques, such as CT, ultrasound or MRI. Additionally, it is possible to distinguish between schwannomas and other tumors, for example, fibrosarcomas or liposarcomas (7), via imaging, and clinical data, such as the size, exact location, association with other organs and invasion can be obtained (8). It has been reported that, to a certain extent, MRI is more sensitive than CT, although they each have the same difficulty in distinguishing bladder schwannoma from carcinoma (3).

In the present case, CT was used to find the exact location of the mass beside the rectum, which was compressed as a result, although there were no symptoms of this. A clear tunica could also be observed, therefore the surgical risk and procedural design could be determined.

During the surgery, incisions were made in the skin and subcutaneous tissue. The mass was confirmed to be in the subcutaneous tissue, as previously shown by CT. Due to the pre-operative examination and assessment, the mass could be completely resected, and the duration of the surgery was not excessive.

Histologically, schwannomas consist of compact cellular lesions. Antoni A (interlacing and cellular fascicles) and Antoni B (less cellular and myxoid) areas, combined with positive uniform S-100 staining characterize the histological appearance of typical schwannomas (4). Therefore, S-100 immunostaining is extremely useful in the differential diagnosis of schwannoma. The mass reported in the present study exhibited the characteristics typical of a schwannoma (9) and positive S-100 immunostaining.

In conclusion, surgical excision, which can be diagnostic and curative, is the optimal treatment for schwannomas, whether they are benign or malignant. In the present case, a large mass developed, however, there were no resultant symptoms. This was unexpected, as changes, such as altered defecation habits, were anticipated with the development of the tumor. This was just one of the reasons that the nature of the tumor could not be precisely determined prior to the surgery.
